# Development of a simple, low-cost and eurytopic medium based on *Pleurotus eryngii* for lactic acid bacteria

**DOI:** 10.1186/s13568-016-0235-7

**Published:** 2016-09-02

**Authors:** Yancun Zhao, Ying Wang, Zhiwei Song, Chengjun Shan, Runjie Zhu, Fengquan Liu

**Affiliations:** 1 Institute of Plant Protection, Jiangsu Academy of Agricultural Sciences, Nanjing, 210014 China; 2 Institute of Agro-product Processing, Jiangsu Academy of Agricultural Sciences, Nanjing, 210014 China

**Keywords:** Lactic acid bacteria, *Pleurotus eryngii*, Nutrient requirements, Sodium acetate, Riboflavin

## Abstract

Lactic acid bacteria (LAB) are a group of important beneficial microorganisms for human, but their growth is restricted to the habitats with rich nutrients. In order to develop a simple, low-cost and efficient medium based on the mushroom *Pleurotus eryngii*, this study evaluated the effects of different treatment methods for the mushroom, concentration of the mushroom, buffers, tween 80, MgSO_4_·7H_2_O, MnSO_4_·4H_2_O, CuSO_4_·5H_2_O, riboflavin and ascorbic acid on the growth of *Lactococcus lactis* subsp. *lactis* SLPE1-3. An optimized medium was developed, which was composed of the mushroom at 200 g/L, the buffer sodium acetate at 5 g/L, and riboflavin at 0.5 mg/L. The mushroom was ground, boiled and filtered for the filtrate in advance. In this optimized medium which was named as PSR medium, the population density of SLPE1-3 sharply reached 2.13 × 10^9^ CFU/mL within 18 h of incubation, and still maintained 1.17 × 10^8^ CFU/mL at 120 h. In addition, this study found that 6 kinds of LAB could grow almost well, and maintained high survival in PSR medium compared to M17 or MRS medium, including *Lactococcus lactis* subsp. *lactis*, *Lactobacillus plantarum*, *Lactococcus lactis* subsp. *cremoris*, *Lactobacillus paracasei*, *Pediococcus pentosaceus* and *Lactobacillus rhamnosus*. These results showed that PSR medium was a simple, low cost and eurytopic medium for the cultivation of LAB, and could replace MRS or M17 medium in the food industry, biomedicine and laboratory.

## Introduction

Lactic acid bacteria (LAB) are a diverse group of Gram-positive, nonsporulating, low G + C content bacteria that include cocci and bacilli, such as *Lactococcus*, *Lactobacillus*, *Streptococcus*, *Pediococcus* and *Leuconostoc* (D’Souza et al. 2012; Wyszyńska et al. 2015). Many bacteria of this group are long known as starters of dairy, plant, and meat fermentations, and could improve the taste and texture of the fermented foods (Price et al. 2012). Thus they are usually retained as beneficial microorganisms for human and animal (Wyszyńska et al. 2015). But it is worthwhile to mention that some strains of LAB could also cause serious diseases in neonates, aquatic animals and postharvest mushroom (Wang et al. 2008; Daniel et al. 2011; Zhao et al. 2013).

During the last two decades, LAB are extensively studied and used in food technology and biomedicine because of their commercial potential, especially *Lactococcus lactis* and some species of the *Lactobacillus* genus (Aller et al. 2014; Wyszyńska et al. 2015). But these microorganisms are fastidious in nutrient requirements, and their growth is restricted to the habitats with rich nutrients (van Niel and Hahn-Hägerdal 1999; Price et al. 2012). During the long process of evolution, LAB lost a number of genes for the biosynthesis of cofactors, and lack various biosynthetic pathways for important nutrients, especially amino acids and vitamins (van Niel and Hahn-Hägerdal 1999; Kelly et al. 2010). Therefore, most of LAB required rich nutrients for their well growth. It is very difficult to develop a generally applicable defined medium for these organisms. Currently, the rich and undefined media are extensively used for culturing LAB, such as MRS medium (De Man et al. 1960) and M17 broth (Terzaghi and Sandine 1975). But these media are composed of diversiform materials, and their prices are very high, such as yeast extract, peptone, polypeptone, soy peptone, beef extract.

*Pleurotus eryngii* is a kind of favored oyster mushroom, and contains rich nutrients, such as protein, fiber, carbohydrates, vitamins and minerals (Cohen et al. 2002; Li and Shah 2015). Li and Shah (2015) found that polysaccharide extracted from *P. eryngii* could promote the proliferation of *Streptococcus thermophilus* in fermented milk, and enhance its viability rate during refrigerated storage at 4 °C (Li and Shah 2015). In the previous study, we found that *L. lactis* subsp. *lactis* could quickly proliferate on the surface of post-harvest *P. eryngii*, and cause water-soaked lesions (Zhao et al. 2013).

Currently, *P. eryngii* has been extensively cultivated in the world (Zhao et al. 2013; Li and Shah 2015). During the cultivation, there is plentiful low-cost inferior mushroom. The objective of this research was to develop a low-cost, simple and eurytopic medium based on the inferior *P. eryngii*, which could replace MRS or M17 broth medium for cultivating various LAB.

## Materials and methods

### Bacterial strains and growth conditions

All bacterial strains used in this study and their origin are listed in Table 1. Six strains of them were isolated from different habitats by our laboratory, including *L. lactis* subsp. *lactis* SLPE1-3, *Lactobacillus plantarum* P13, *Streptococcus thermophilus* M1-6, *Lactobacillus paracasei* FM-LP-4, *Leuconostoc mesenteroides* JX5 and *Pediococcus pentosaceus* SR2-6. *L. lactis* subsp*. cremoris* MG1363 was donated by Prof. Lixin Luo (South China University of Technology, China). *Lactobacillus rhamnosus* GG was purchased from American Type Culture Collection (ATCC, America). Unless otherwise stated, all bacterial strains were grown at 30 °C for 18 h in MRS medium (proteose peptone at 10 g/L, beef extract at 10 g/L, yeast extract 5 g/L, dextrose 20 g/L, polysorbate 80 1 mL/L, ammonium citrate 2 g/L, sodium acetate 5 g/L, magnesium sulfate 0.1 g/L, manganese sulfate 0.05 g/L, dipotassium phosphate 2 g/L, pH 6.8). The bacterial culture was used as inoculum in the following experiments.

**Table 1:** Lactic acid bacteria (LAB) strains used in this study

Strain	Origin	Source or reference
*Lactococcus lactis* subsp. *lactis* SLPE1-3 (CGMCC12634)	*Pleurotus eryngii*	This laboratory
*Lactobacillus plantarum* P13	Fermented vegetables	This laboratory
*Lactococcus lactis* subsp*. cremoris* MG1363	Plasma cured strain NCDO 712	Jensen and Hammer (1993)
*Streptococcus thermophilus* M1-6	Dairy	This laboratory
*Lactobacillus paracasei* FM-LP-4 (CGMCC8600)	Dairy	This laboratory
*Pediococcus pentosaceus* SR2-6	Sour meat	This laboratory
*Lactobacillus rhamnosus* GG (ATCC53103)	Human gastrointestinal tract	Gorbach (1996)
*Leuconostoc mesenteroides* JX5	Kefir	This laboratory

### Effect of different treatment methods for *P. eryngii* on the growth of *L. lactis* subsp. *lactis*

In this study, the fresh mushroom *P. eryngii* was bought from a super vegetable wholesale market in Nanjing, Jiangsu Province, China. The mushroom was cut into small pieces (1.5–2.0 cm^3^) for this experiment. This experiment included three treatments: (1) 200 g of the mushroom was ground in a blender (MJ-M176P, Panasonic Limited, Malaysia) for 3 min at high speed with 400 mL water. The mushroom slurry was supplemented with 500 mL water, boiled for 20 min, and then filtered through two layers of medical gauze. The final volume of the filtrate was increased to 1000 mL by supplemented water; (2) 200 g of the mushroom was ground according to the above description, and then filtered through two layers of medical gauze. The final volume of the filtrate was increased to 1000 mL by supplemented water; (3) 200 g of the mushroom were boiled for 20 min, and then filtered through two layers of medical gauze. The final volume of the filtrate was increased to 1000 mL by supplemented water. The pH values were adjusted to 6.8 ± 0.1, respectively. These media were sterilized for 20 min at 121 °C.

Subsequently, 250 μL of *L. lactis* subsp. *Lactis* SLPE1-3 culture was added to 50 mL of the above-mentioned filtrate media respectively, and incubated for 72 h at 30 °C without shaking. The population dynamics of SLPE1-3 was investigated after 0, 3, 9, 18, 30, 48 and 72 h of incubation by the gradient dilution method. In brief, each bacterial culture was diluted with sterile water, and the diluents were plated on MRS agar plates respectively. The number of bacterial colonies on each plate was counted after incubating for 24 h at 30 °C. Every treatment included three repetitions.

### Effect of different concentration of *P. eryngii* on the growth of *L. lactis* subsp. *lactis*

According to the description in the above section, 100, 150, 200 and 250 g of the mushroom was respectively ground to make mushroom slurry, boiled for 20 min, and then filtered. The final volume of each filtrate was increased to 1000 mL by supplemented water. The pH values were initially adjusted to 6.8 ± 0.1, respectively. These media were sterilized for 20 min at 121 °C. Subsequently, 250 μL of *L. lactis* subsp. *lactis* SLPE1-3 culture was added to 50 mL of the filtrate media respectively, and incubated for 72 h at 30 °C without shaking. The population dynamics of SLPE1-3 was investigated after 0, 9, 18, 30, 48 and 72 h of incubation by the gradient dilution method. Every treatment included three repetitions.

### Effect of different buffers on the growth of *L. lactis* subsp. *lactis*

According to the description in the above section, 1000 g of the mushroom was ground to make mushroom slurry, boiled for 20 min, and then filtered. The filtrate was equally divided into five parts. Three kinds of buffers, including to ammonium citrate, sodium acetate and dipotassium phosphate, were selected and supplemented based on MRS medium (De Man et al. 1960). This experiment included five treatments: (1) the filtrate was supplemented with ammonium citrate at a final concentration of 2 g/L; (2) the filtrate was supplemented with sodium acetate at a final concentration of 5 g/L; (3) the filtrate was supplemented with dipotassium phosphate at a final concentration of 2 g/L; (4) the filtrate was supplemented with ammonium citrate at 2 g/L, sodium acetate at 5 g/L, and dipotassium phosphate at 2 g/L; (5) the filtrate without buffer was as the control. The final volume of each filtrate was increased to 1000 mL by supplemented water. The pH values were initially adjusted to 6.8 ± 0.1, respectively. These media were sterilized for 20 min at 121 °C.

Subsequently, 250 μL of *L. lactis* subsp. *lactis* SLPE1-3 culture was added to 50 mL of the above-mentioned filtrate media, respectively, and incubated for 72 h at 30 °C without shaking. The population dynamics of SLPE1-3 was investigated after 0, 9, 18, 30, 48 and 72 h of incubation by the gradient dilution method. Every treatment included three repetitions.

### Effect of different additives on the growth of *L. lactis* subsp. *lactis*

In this experiment, 200 g of the mushroom was ground to make mushroom slurry, boiled for 20 min, and then filtered. The filtrate was supplemented with sodium acetate at a final concentration of 5 g/L. The final volume of the filtrate was increased to 1000 mL by supplemented water. The seven kinds of additives were added respectively to the filtrate at the following final concentrations: glucose 5 g/L, tween 80 0.5 ml/L, MgSO_4_·7H_2_O 0.2 g/L, MnSO_4_·4H_2_O 20 mg/L, CuSO_4_·5H_2_O 3 mg/L, riboflavin 0.5 mg/L, ascorbic acid 0.5 mg/L. These additives were selected based on their previously reported influences on the growth of *L. lactis* (De Man et al. 1960; van Niel and Hahn-Hägerdal 1999; Aller et al. 2014). The filtrate without additive was as the control. The pH values were initially adjusted to 6.8 ± 0.1, respectively. These media were sterilized for 20 min at 121 °C.

Subsequently, 250 μL of *L. lactis* subsp. *lactis* SLPE1-3 culture was added to 50 mL of the above-mentioned filtrate media, respectively, and incubated for 120 h at 30 °C without shaking. The population dynamics of SLPE1-3 was investigated after 0, 9, 18, 30, 48, 72 and 120 h of incubation by the gradient dilution method. Every treatment included three repetitions.

### Growth dynamics of eight LAB strains in PSR, MRS and M17 media

Based on the above experimental results, the optimized medium was composed of the mushroom filtrate at 200 g/L, sodium acetate at 5 g/L, and riboflavin at 0.5 mg/L. This medium was named as PSR medium. The pH value was adjusted to 6.8 ± 0.1. Then the medium was sterilized for 20 min at 121 °C.

Subsequently, 250 μL of each LAB strain culture was added to 50 mL of PSR, MRS or M17 medium (Hope Bio-Technogy, Qingdao, China) respectively, and incubated for 120 h at 30 °C without shaking. The population dynamics of each LAB strain was investigated after 0, 3, 9, 18, 30, 48, 72 and 120 h of incubation by the gradient dilution method. Every treatment included three repetitions.

### Statistical analysis

Every treatment was randomly arranged with three replicates. All data obtained were subjected to analysis of variance (ANOVA) using SPSS 13.0 software (SPP Inc., Chicago, USA). The mean values were compared using Tukey’s test at *P* < 0.05.

## Results

### Effect of different treatment methods for *P. eryngii* on the growth of *L. lactis* susp. *lacti*s

**Fig. 1:**
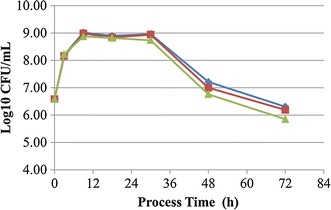
Effect of different treatment methods for *Pleurotus eryngii* mushroom on the growth of *Lactococcus lactis* subsp. *lactis* SLPE1-3. *Filled diamond* the mushroom was ground in a blender for 3 min, boiled for 20 min, and then filtered; *filled square* the mushroom was ground in a blender for 3 min, and then filtered; *filled triangle* the mushroom was boiled for 20 min, and then filtered. Each *symbol* is the average of three replicates

In three different treatments for *P. eryngii*, the growth of *L. lactis* susp. *lactis* SLPE1-3 showed a similar trend (Fig. 1). The cell density increased sharply within 9 h of incubation, kept a high level from 9 to 30 h of incubation, and then decreased rapidly after 30 of inoculation. But the mushroom *P. eryngii* medium, which was ground and boiled, could improve survival probability compared to other treatment methods at the late stage of incubation. Hence, the mushroom *P. eryngii* was ground, boiled and filtered for the subsequent experiments.

### Effect of different concentration of *P. eryngii* on the growth of *L. lactis* subsp. *lactis*

**Fig. 2:**
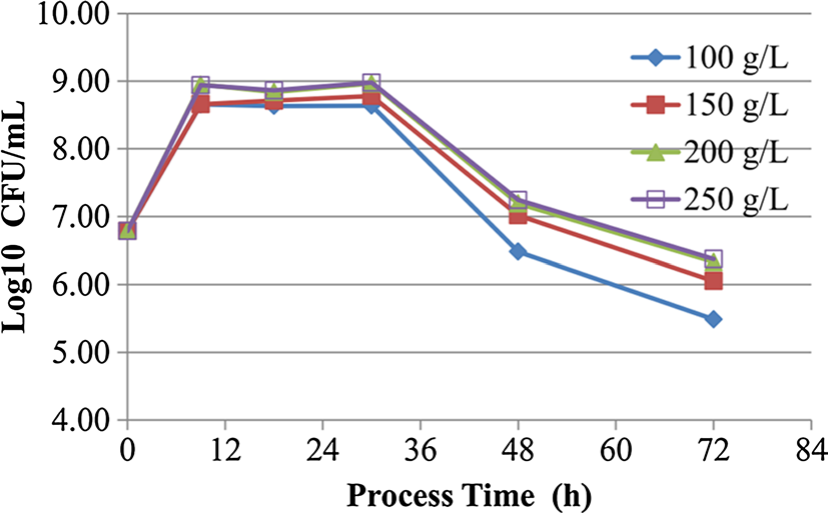
Effect of different concentration of *P. eryngii* mushroom on the growth of *L. lactis* subsp. *lactis* SLPE1-3. Each *symbol* is the average of three replicates

In this experiment, there was a positive relationship between the cell density of *L. lactis* subsp. *lactis* SLPE1-3 and the concentration of *P*. *eryngii* (<200 g/L), especially at the late stage (Fig. 2). But there was not remarkably difference between 200 and 250 g/L of *P. eryngii*. Hence, 1000 mL medium contained 200 g of the mushroom *P. eryngii* in the following experiments.

### Effect of different buffers on the growth of *L. lactis* subsp. *lactis*

**Fig. 3:**
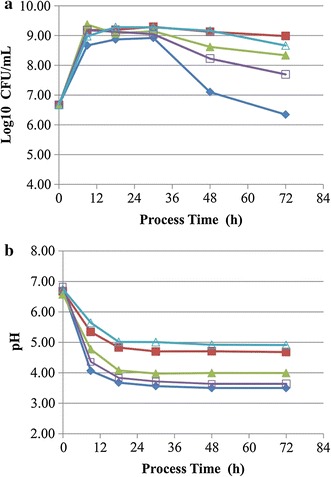
Effect of different buffers on the growth of *L. lactis* subsp. *lactis* SLPE1-3 (**a**) and the pH values of the culture media (**b**). *Filled diamond* the control without buffer; *filled square* sodium acetate at 5 g/L; *filled triangle* ammonium citrate at 2 g/L; *open square* dipotassium phosphate at 2 g/L; *open triangle* sodium acetate at 5 g/L, ammonium citrate at 2 g/L and dipotassium phosphate at 2 g/L. Each *symbol* is the average of three replicates

MRS medium, which was extensively used for culturing various LAB species, contained three kinds of buffers, including ammonium citrate, sodium acetate and dipotassium phosphate. When these buffers were supplemented alone or jointly to the *P. eryngii* medium, the population density of *L. lactis* susp. *lactis* SLPE1-3 was significantly higher than that of the control without any buffer, especially alone sodium acetate at the late stage of incubation (Fig. 3a). At 72 h of incubation, the viable count in the medium supplemented with sodium acetate (5 g/L) was 9.67 × 10^8^ CFU/mL, which was 403.9 times that of the control. In addition, alone sodium acetate or the jointly buffers showed higher buffer capacity than ammonium citrate and dipotassium phosphate (Fig. 3b). Hence, sodium acetate was used as the buffer in the following experiments.

### Effect of different additives on the growth of *L. lactis* subsp. *lactis*

**Fig. 4:**
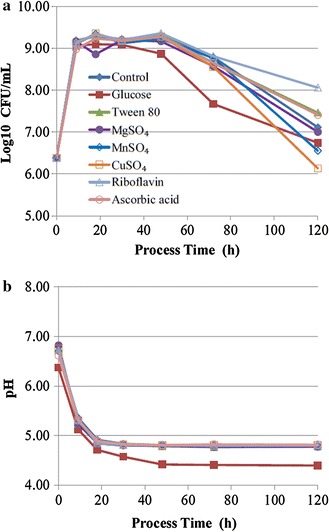
Effects of 7 kinds of additives on the growth of *L. lactis* subsp. *lactis* (**a**) and the pH values of the culture media (**b**). Each *symbol* is the average of three replicates

In this experiment, we investigated the effect of seven additives on the growth of *L. lactis* subsp. *lactis* SLPE1-3. The results showed that these additives could not promote the growth of SLPE1-3 within 72 h after inoculating compared to the control without additives (Fig. 4a). After 48 h of incubation, the population densities of SLPE1-3 sharply decreased in the media supplemented difference additives, especially glucose, MnSO_4_ and CuSO_4_ (Fig. 4a). But the additives tween 80, riboflavin and ascorbic acid could remarkably delay the contabescence of SLPE1-3 compared to the control, especially riboflavin. At 120 h of incubation, the population density of SLPE1-3 in the medium supplemented riboflavin was 8.88 times that of the control. In addition, the additive glucose prevented the growth of SLPE1-3 at the earlier stage compared to the control, and promoted its contabescence at the later stage. Among seven additives, only glucose significantly promoted the decrease in the pH value of the culture medium compared to the control (Fig. 4b). The lower pH value might be disadvantageous to the growth and viability of LAB.

**Fig. 5:**
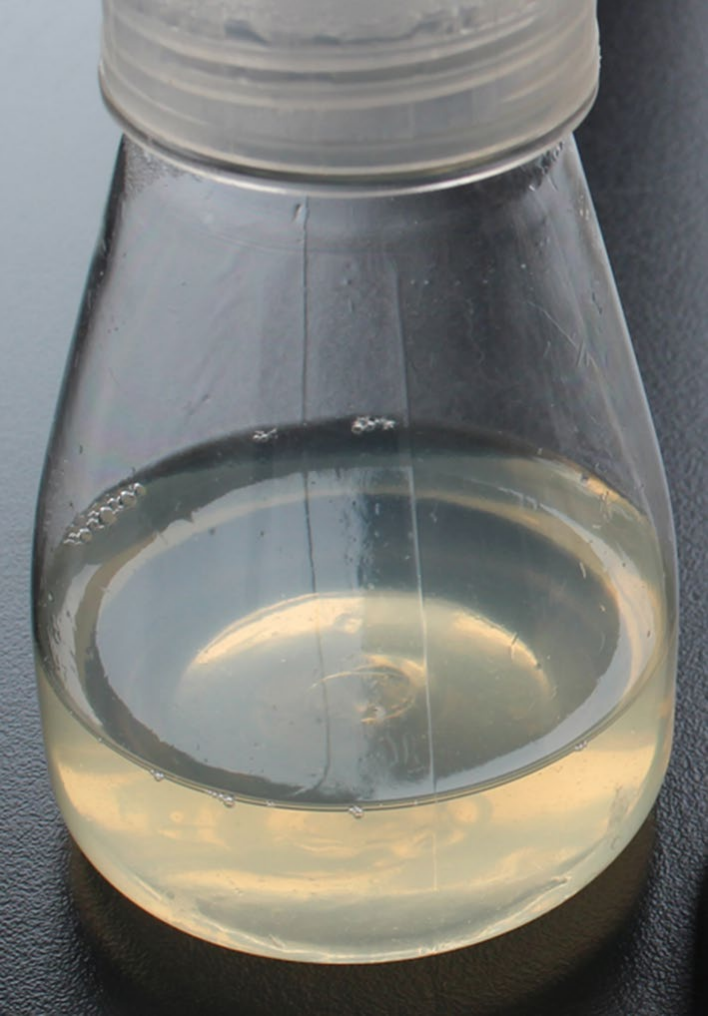
Physical form of the liquid PSR medium which was composed of *Pleurotus eryngii* mushroom at 200 g/L, sodium acetate at 5 g/L, and riboflavin at 0.5 mg/L. The mushroom was ground to make mushroom slurry, boiled for 20 min, and then filtered for the filtrate in advance

Based on these results, the optimized medium was composed of the mushroom *P. eryngii* at 200 g/L, sodium acetate at 5 g/L, and riboflavin at 0.5 mg/L, which was named as PSR medium. In addition, the physical property of PSR medium was relatively stable, and there was not obvious sedimentation and layer phenomena within 30 days of storage at room temperature (Fig. 5).

### Growth dynamics of eight LAB strains in PSR, MRS and M17 media

**Fig. 6:**
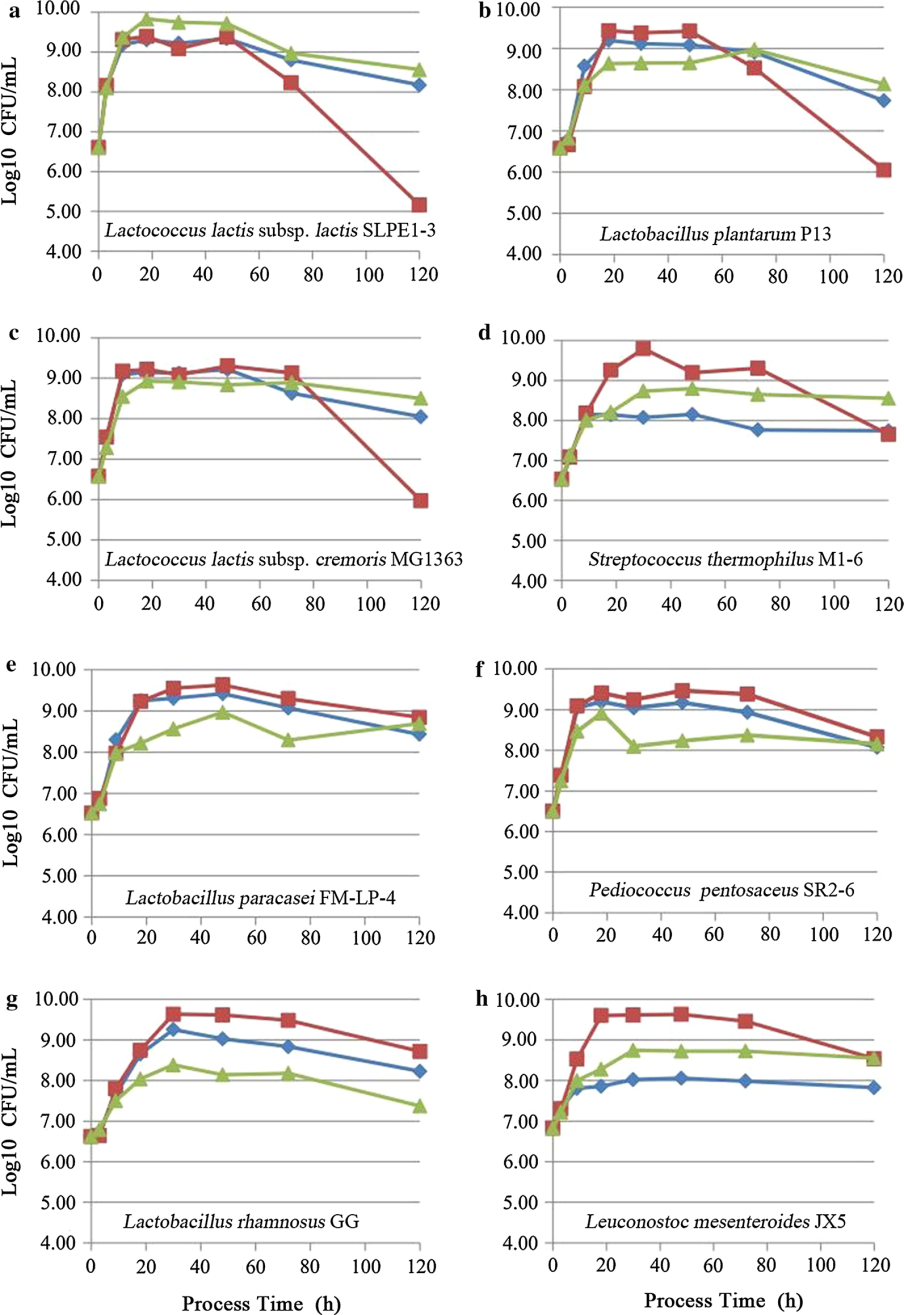
Growth dynamics of eight lactic acid bacteria strains in PSR, MRS and M17 media. **a** *Lactococcus lactis* subsp. *lactis* SLPE1-3; **b** *Lactobacillus plantarum* P13; **c** *Lactococcus lactis* subsp. *cremoris* MG1363; **d** *Streptococcus thermophilus* M1-6; **e** *Lactobacillus paracasei* FM-LP-4; **f** *Pediococcus pentosaceus* SR2-6; **g** *Lactobacillus rhamnosus* GG; **h** *Leuconostoc mesenteroides* JX5. *Filled diamond* PSR medium which was composed of *P. eryngii* mushroom at 200 g/L, sodium acetate at 5 g/L, and riboflavin at 0.5 mg/L; *filled square* MRS medium; *filled triangle* M17 medium. Each *symbol* is the average of three replicates

To examine the suitability of PSR medium for various LAB, a total of 8 LAB strains, belonging to seven species, were tested for their growth in PSR, MRS and M17 media. Three strains of them (*L. lactis* subsp. *lactis* SLPE1-3, *L. plantarum* P13, *L. lactis* subsp. *cremoris* MG1363) grew almost as well and remained highly survival rates in PSR and M17 media, but their population densities were sharply decreased after 48 h of incubation in MRS medium (Fig. 6a–c). Two strains of them (*L. paracasei* FM-LP-4, *Pediococcus pentosaceus* SR2-6) grew almost as well in PSR and MRS medium, and better compared to M17 medium (Fig. 6e, f). One strain of them (*Lactobacillus rhamnosus* GG) grew better in PSR medium compared to M17 medium, but poorly compared to MRS medium (Fig. 6g). In addition, two strains of them (*S. thermophilus* M1-6, *L. mesenteroides* JX5) grew poorly in PSR compared to MRS and M17 media (Fig. 6d, h). These results showed that PSR medium had high suitability for the cultivation of many LAB.

## Discussion

The previous studies had demonstrated that 6-8 kinds of amino acids must be supplemented in chemically defined media (CDM) for the cultivation of various LAB (Jensen and Hammer 1993; Cocaign-Bousquet et al. 1995; Aller et al. 2014). Among these amino acids, glutamic acid and asparagine were the most important media components for the growth of many LAB (Aller et al. 2014). In addition, LAB could grow better in the medium containing 18-19 kinds of amino acids (van Niel and Hahn-Hägerdal 1999). The mushroom *P. eryngii* contains rich protein and free amino acids, especially aspartic acid, glutamic acid and arginine (Stajić et al. 2009). This study founded that 6 kinds of LAB could grow very well in PSR medium when the mushroom *P. eryngii* was used as only nitrogen source, and their population densities were more than 1 × 10^9^ CFU/mL at 30 h of incubation. This study demonstrated that the mushroom *P. eryngii* could supply enough amino acids for the growth of most species of LAB.

In bioprocesses, the pH value is an important environmental factor. The influence of pH on the growth and metabolic processes of LAB had been extensively studied (van Niel and Hahn-Hägerdal 1999; Aller et al. 2014). The optimum pH for the growth of LAB was usual between 6.0 and 6.5. But, LAB produced much lactic acid during fermentation, which led to a sharp drop of the pH of the culture medium (Wyszyńska et al. 2015). The growth of LAB was inhibited when the pH of the culture medium was under 5.5 (van Niel and Hahn-Hägerdal 1999). The previous study had demonstrated that some buffers could improve the growth and viability of LAB, such as sodium acetate, ammonium citrate, dipotassium phosphate (De Man et al. 1960; Aller et al. 2014). In this study, we found that sodium acetate was a more suitable buffer for the growth and viability of *L. lactis* susp. *lactis* SLPE1-3 than ammonium citrate and dipotassium phosphate. Sodium acetate showed higher buffering ability for the pH of the culture medium than ammonium citrate and dipotassium phosphate. This study demonstrated again the lower pH of the culture medium remarkably prevented to the growth LAB, and promoted the death of LAB (Broadbent et al. 2010).

Besides amino acids and pH value, many studies had demonstrated that several B-group vitamins were essential for the growth of LAB (van Niel and Hahn-Hägerdal 1999; Aller et al. 2014). Aller et al. (2014) founded that riboflavin (B2) was the only essential B-group vitamin for the growth of *L. lactis* IL1403 in the chemically defined media (CDM) experiment. Riboflavin is indispensable for cellular metabolism because it is the precursor of coenzymes flavin mononucleotide (FMN) and flavin adenie dinucleotide (FAD) (LeBlanc et al. 2011; Aller et al. 2014). The mushroom *P. eryngii* contains significant concentrations of vitamins, including C, A, B_2_, B_1_, D and niacin (Manzi et al. 1999; Stajić et al. 2009). Hence, the PSR medium based on the mushroom *P. eryngii* contains rich vitamins. In this study, though the additive riboflavin could not further promote the growth of *L. lactis* susp. *lactis* SLPE1-3, it delayed the decay of SLPE1-3 in the culture medium compared to the control without the additive riboflavin.

Rich media MRS and M17 contain adequate amounts of minerals through the use of yeast extract, such as Fe^2+^, Cu^2+^, Mg^2+^, Zn^2+^, Ca^2+^, etc. (van Niel and Hahn-Hägerdal 1999). Some studies founded that Mg^2+^, Mn^2+^ and Cu^2+^ could stimulate the growth of LAB (Olsen and Qutub 1970; Hansson and Häggström 1984; Loubiere et al. 1997; Aller et al. 2014). The mushroom *P. eryngii* contains rich mineral elements, especially Mg, Cu, Mn, K and Ca (Stajić et al. 2009; Akyüz and Kirbag 2010). In this study, the supplementation of mineral elements (MgSO_4_, MnSO_4_ and CuSO_4_) could not simulate the growth of *L. lactis* susp. *lactis* SLPE1-3. This result showed that the PSR medium based on the mushroom *P. eryngii* contained sufficient Mg, Mn and Cu, and excessive mineral elements were not conducive to the growth and viability of LAB.

Since LAB have been extensively used in food technology, biomedicine and scientific experiments, some researchers committed themselves to developing a simple and low-cost medium for the growth of various LAB (De Man et al. 1960; Terzaghi and Sandine 1975; Rodriguez et al. 2010; Aller et al. 2014). During the fermentation of *L. lactis* CECT-4434 for producing lactic acid and biosurfactant, MRS medium could be replaced with two waste materials: trimming vine shoots as C source, and distilled wine lees as N, P and micronutrient sources (Rodriguez et al. 2010). In this study, PSR medium was only composed of the low-cost mushroom *P. eryngii*, sodium acetate and riboflavin. The cost of raw materials is only 0.8 dollar or so for 1 L of PSR medium in China, but about 2.5 dollars for 1 L of MRS or M17 medium. PSR medium could replace MRS or M17 medium for the cultivation of *L. lactis* subsp. *lactis*, *L. plantarum*, *L. lactis* subsp. *cremoris*, *L. paracasei*, *P. pentosaceus*, *L. rhamnosus*, etc.

In conclusion, this study developed a kind of simple, low-cost and eurytopic medium (PSR medium). Compared to MRS or M17 medium, many LAB could grow almost as well, and remain highly survival rates in PSR medium. Hence, PSR medium could replace MRS or M17 medium for the cultivation of many LAB in the food industry, biomedicine and laboratory.

## Abbreviations

LAB: lactic acid bacteria
CGMCC: China General Microbiological Culture Collection Center
ATCC: American Type Culture Collection
CDM: chemically defined media
FMN: flavin mononucleotide
FAD: flavin adenie dinucleotide
ANOVA: analysis of variance
